# Temperature-Responsive Polysaccharide Microparticles Containing Nanoparticles: Release of Multiple Cationic/Anionic Compounds

**DOI:** 10.3390/ma15134717

**Published:** 2022-07-05

**Authors:** Takumi Sato, Yoshihiko Murakami

**Affiliations:** Department of Organic and Polymer Materials Chemistry, Tokyo University of Agriculture and Technology, Naka-cho, Koganei-shi 2-24-16, Tokyo 184-8588, Japan; takumi.sato.2021@gmail.com

**Keywords:** polysaccharide, carrageenan, nanoparticle, pulmonary drug delivery

## Abstract

Most drug carriers used in pulmonary administration are microparticles with diameters over 1 µm. Only a few examples involving nanoparticles have been reported because such small particles are readily exhaled. Consequently, the development of microparticles capable of encapsulating nanoparticles and a wide range of compounds for pulmonary drug-delivery applications is an important objective. In this study, we investigated the development of polysaccharide microparticles containing nanoparticles for the temperature-responsive and two-step release of inclusions. The prepared microparticles containing nanoparticles can release two differently charged compounds in a stepwise manner. The particles have two different drug release pathways: one is the release of nanoparticle inclusions from the nanoparticles and the other is the release of microparticle inclusions during microparticle collapse. The nanoparticles can be efficiently delivered deep into the lungs and a wide range of compounds are released in a charge-independent manner, owing to the suitable roughness of the microparticle surface. These polysaccharide microparticles containing nanoparticles are expected to be used as temperature-responsive drug carriers, not only for pulmonary administration but also for various administration routes, including transpulmonary, intramuscular, and transdermal routes, that can release multiple drugs in a controlled manner.

## 1. Introduction

A variety of nanoparticles have been developed, including inorganic nanoparticles formed from silica and gold and polysaccharide nanoparticles formed from alginate and chitosan [1,2,3,4], as drug carriers in various drug-dosage forms, including oral and intravenous administration [5,6,7]. Among these, cationic nanoparticles have been reported to exhibit antimicrobial activities and membrane permeabilities, which can be used as anticancer agents [8,9]. Biologically-derived polymers, such as chitosan and poly-l-lysine, are often used as cationic polymers to form cationic nanoparticles [10,11,12]. Chitosan is a linear polymer prepared by deacetylating natural chitin, which is obtained from crustaceans, such as shrimps and crabs. Poly-l-lysine is a polymer produced by the fermentation of lysine by *Streptomyces albulus* [12]. Nanoparticles formed from chitosan or poly-l-lysine have been reported on several occasions [12,13,14,15]. For example, mixing chitosan or poly-l-lysine with tripolyphosphoric acid (TPP) has been reported to form nanoparticles through electrostatic interactions between the amino groups of the cationic polymer and phosphate groups of TPP [16,17,18,19]; such nanoparticles are easily formed by ultrasonication in the absence of surfactants.

Pulmonary administration [20,21], in which drugs are inhaled and absorbed into the body through the lungs, is a medication method that has several advantages, including simplicity of administration [22], excellent immediate efficacy [23], and efficient treatment of lung diseases [24,25]. However, current pulmonary administration uses drug carriers that are mainly microparticles with diameters over 1 µm; only a few examples have used nanoparticles because nanoparticles less than 1 µm in diameter are too small for pulmonary administration. This is because they are expelled from the body through exhalation [26].

We previously developed temperature-responsive carrageenan microparticles that are efficiently delivered to the lungs [27]. These particles, which are readily prepared by the sol–gel transition of carrageenan, can rapidly release their inclusions by collapsing in response to temperature. Carrageenan particles are formed from water-in-oil (w/o) emulsions and readily encapsulate the compounds dissolved or dispersed in the carrageenan solution. However, as carrageenan particles are negatively charged, encapsulating negatively charged compounds is difficult owing to electrostatic repulsion. Therefore, developing carrageenan microparticles capable of encapsulating a wide range of single or multiple compounds is important.

**Figure 1:**
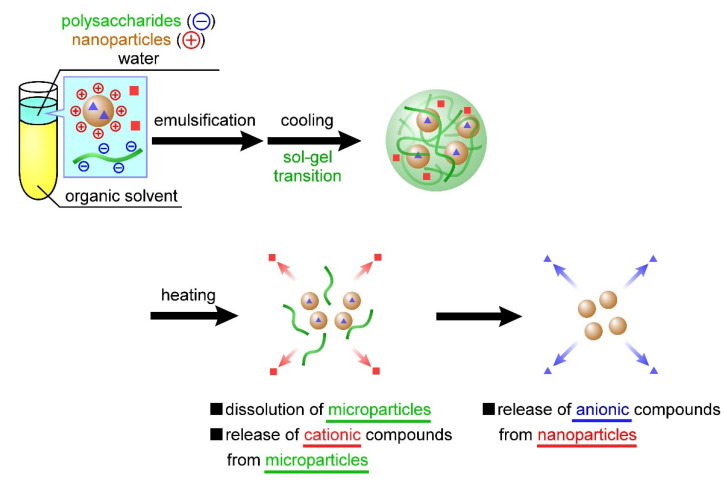
Temperature-responsive nanoparticle-containing or nanoparticle-decorated polysaccharide microparticles for the release of multiple compounds. The nanoparticles and compounds (red squares) dispersed inside the microparticles are released in a temperature-responsive manner, followed by the release of another compound (blue squares) from within the nanoparticles.

In the present study, we developed temperature-responsive microparticles by complexing cationic nanoparticles with anionic microparticles. Dispersing these cationic nanoparticles in an aqueous carrageenan solution during microparticle preparation facilitates their complexation with anionic microparticles through electrostatic interactions. Furthermore, these cationic nanoparticles can contain anionic compounds, also through electrostatic interactions. Therefore, the previously developed carrageenan microparticles [27] can contain positively charged compounds, whereas the microparticles developed in this study facilitate the concurrent containment of negatively charged compounds through complexation with cationic nanoparticles. The carrageenan microparticles designed based on this idea should exhibit two-step release behavior; that is, the nanoparticles and compounds dispersed inside the microparticles are released in a temperature-responsive manner, followed by the release of another compound from within the nanoparticles (Figure 1). In the present study, the nanoparticles were first prepared using chitosan or poly-l-lysine, after which we evaluated the release behavior of the negatively charged compounds from these nanoparticles. Using the sol–gel transition of carrageenan, we then investigated the complexation of the nanoparticles in the microparticles. In addition, we prepared carrageenan microparticles containing both positively charged compounds and the aforementioned nanoparticles and evaluated the release behavior of the two compounds from the microparticles. To the best of our knowledge, the release of a wide range of compounds by combining polysaccharide microparticles and biopolymer nanoparticles based on the proposed technology that controls both the sol–gel transition and emulsion formation has not been reported.

## 2. Materials and Methods

### 2.1. Materials

κ-Carrageenan (κ-CRG), potassium chloride, methylene blue trihydrate (Mb), pentasodium triphosphate (TPP), acetic acid, hydrochloric acid, sodium hydroxide solution, and toluene were purchased from Wako Pure Chemical Industries (Osaka, Japan). ι-Carrageenan (ι-CRG) and poly-l-lysine (405 kDa, PLL) were purchased from Sigma-Aldrich (St. Louis, MO, USA). Sodium 2-naphthalenesulfonate (Ns) was purchased from Tokyo Chemical Industry Co., Ltd. (Tokyo, Japan). Chitosan (100 kDa, CS) was obtained from Dainichiseika Color & Chemicals Mfg. Co., Ltd. (Tokyo, Japan). Poly(ethylene glycol)-*b*-poly(ε-caprolactone) block copolymer (PEG-*b*-PCL) was synthesized as a polymeric surfactant according to a previously reported method [28,29] with slight modifications (*M*_n_s of the PEG and PCL units were 3500 and 4300, respectively; *M*_w_/*M*_n_ ratios of PEG and PEG-*b*-PCL were 1.09 and 1.35, respectively). All other reagents were of analytical grade and used without further purification. The chemical structures of κ-CRG, ι-CRG, CS, PLL, TPP, Mb, and Ns are shown in Figure S1.

### 2.2. Preparation of CS(Ns) and PLL(Ns) Nanoparticles

The CS(Ns) and PLL(Ns) nanoparticles were prepared according to previously reported methods [16,18,19,30,31,32]. Here, *A*(*m*) nanoparticles refer to nanoparticles *A* that contain *m* inside them. If a compound is contained within the nanoparticles, then *m* is the name of the compound; otherwise, *m* is simply written as “-” if the nanoparticle has no compound. CS(Ns) nanoparticles were prepared by dropping an aqueous solution (5 mL, pH 5) of TPP (4.2 mg) into an acetate buffer solution (10 mL, pH 5) containing CS (20 mg) and Ns (20 mg) under sonication (20 kHz, 5 min) with an ultrasonic homogenizer (UH-50, SMT Co., Ltd., Tokyo, Japan). The solution was ultrasonicated for another 10 min, then stirred at 300 rpm for 30 min and centrifuged at 9200 rpm for 20 min to produce CS(Ns) nanoparticles. In contrast, PLL(Ns) nanoparticles were prepared by dropping an aqueous solution (5 mL, pH 4) containing TPP (0.92 mg) into an aqueous solution (10 mL, pH 4) of PLL (10 mg) and Ns (20 mg) under sonication (20 kHz, 5 min) with an ultrasonic homogenizer. The pH was then adjusted to 7 with NaOH, and then ultrasonicated for another 10 min. The PLL(Ns) nanoparticles were subsequently obtained following the same procedure used for the CS(Ns) nanoparticles.

### 2.3. Preparation of the CRG(CS(Ns), Mb) and CRG(PLL(Ns), Mb) Microparticles

The CRG(CS(Ns), Mb) and CRG(PLL(Ns), Mb) microparticles were prepared according to a previously reported method [27]. Here, *A*(*m*, *n*) refers to microparticle *A* that contains nanoparticle *m* and compound *n* inside them. If the microparticle contains only one nanoparticle or compound, then only *m* is provided, whereas *m* is simply written as “-” if the microparticle contains no compound or nanoparticle. An aqueous polysaccharide solution was prepared by dissolving κ-CRG (2.5 *w*/*v*%), ɩ-CRG (2.5 *w*/*v*%), potassium chloride (32 mM), and Mb (7.3 mM) in Milli-Q water (70 °C, 40 mL). A mixture of κ-CRG and ι-CRG was used instead of κ-CRG or ι-CRG alone because the mixture gelates rapidly with moderate rigidity [27]. The organic solvent was prepared by dissolving PEG-*b*-PCL (0.5 mM) in toluene (10 mL). The w/o emulsion was then prepared by emulsifying (70 °C, 12,000 rpm, 5 min) the polysaccharide solution (0.35 g), organic solvent, and nanoparticle dispersion (1.6 mg/mL CS(Ns) or 0.6 mg/mL PLL(Ns) nanoparticles, 100 µL) with a high-speed homogenizer (NS-51 K and NS-10, Microtec Co., Ltd., Chiba, Japan). The emulsion was gradually cooled to 25 °C in a water bath, and the microparticles were washed three times by the repetitive addition of toluene and subsequent centrifugation to remove excess PEG-*b*-PCL. The CRG(CS(Ns), Mb) and CRG(PLL(Ns), Mb) microparticles were finally obtained after drying naturally in a perfluoroalkoxylalkan beaker. For comparison, the formation of the microparticles using only PLL (Ns) nanoparticles without CRG was also investigated. The w/o emulsion was prepared by mixing a solution of PEG-*b*-PCL (0.5 mM) in toluene (10 mL), Milli-Q water (0.35 mL), and nanoparticle dispersion (100 µL) with a homogenizer (70 °C, 12,000 rpm, 5 min). The white solid was collected by the same process used to prepare the CRG-containing microparticles.

### 2.4. Characterization of Nanoparticles and Microparticles

The hydrodynamic diameters of the nanoparticles and microparticles were determined by dynamic light scattering (DLS). A particle dispersion (1 mg/mL) was placed in the DLS instrument (Zetasizer Nano-ZS; Malvern Instruments, Malvern, UK) and the effects of temperature (70 °C) and homogenization (12,000 rpm, 5 min) on the nanoparticle diameter were evaluated. The particle zeta potentials were also determined using the same instrument. All the experiments were performed three times.

The nanoparticle morphologies were examined by transmission electron microscopy (TEM; JEM-1400, JEOL, Tokyo, Japan). A CS(Ns) or PLL(Ns) nanoparticle dispersion (5 µL) was applied to a grid (Microgrid Cu200, JEOL, Tokyo, Japan), and the solution was removed using a filter paper after 1 min. A drop of gadolinium acetate (2.5 *w*/*w*%, 5 µL) was applied for 1 min to negatively stain the sample. The excess staining solution was removed using a filter paper and the sample was dried for 15 min. An accelerator voltage of 120 kV was used for the TEM.

The microparticles morphologies were examined by scanning electron microscopy (SEM; VE-9800, KEYENCE, Osaka, Japan) with an accelerator voltage of 1.3 kV. The specimens were prepared by placing the microparticles on an aluminum plate and coating them with an ~10-nm-thick platinum thin film under reduced pressure using an MSP-1S ion coater (Vacuum Device, Ibaraki, Japan).

The nanoparticle inclusions in the microparticles were examined by differential scanning calorimetry (DSC). The CRG(CS(Ns), Mb) and CRG(PLL(Ns), Mb) microparticles (5 mg) were placed in a sealed aluminum pan and subjected to DSC at 10 °C/min from 0 to 500 °C using a differential scanning calorimeter (DSC-60A plus, SHIMADZU, Kyoto, Japan). To examine in detail the complexation of nanoparticles inside the microparticles, we subjected the following samples to DSC: CS(Ns) and PLL(Ns) nanoparticles, CRG(Mb) microparticles, CS, PLL, Mb, Ns, TPP, PEG-*b*-PCL, and κ,ι-CRG (i.e., a physical mixture of κ-CRG and ι-CRG).

The following quantities were determined by spectrofluorometry (FP-6500, JASCO Co., Ishikawamachi, Japan; *λ*_ex_ = 273 nm, *λ*_em_ = 324 nm for Ns, *λ*_ex_ = 640 nm, *λ*_em_ = 680 nm for Mb): (1) encapsulation ratios and efficiencies of Ns in the CS(Ns) and PLL(Ns) nanoparticles; (2) encapsulation ratios and efficiencies of Mb in the CRG(CS(Ns), Mb) and CRG(PLL(Ns), Mb) microparticles; (3) retention ratio and efficiencies of the CS (Ns) nanoparticles in the CRG(CS(Ns), Mb) microparticles; and (4) retention ratio and efficiencies of the PLL(Ns) nanoparticles in the CRG(PLL(Ns), Mb) microparticles. The nanoparticles and microparticles were placed in a dialysis membrane and immersed in phosphate-buffered saline (PBS). At regular intervals (10 min), the solution outside the dialysis membrane was collected, and its fluorescence intensity was determined by spectrofluorometry. The number of fluorescent substances (Ns or Mb) contained in each particle was determined from the fluorescence intensity at saturation. The encapsulation rate and efficiencies of Ns for the nanoparticles and Mb for the microparticles were calculated using the amounts of particles recovered, fluorescent substance used in the preparation of the particles, and fluorescent substance contained in the particles. The retention ratio and efficiencies of the nanoparticles in the microparticles were calculated based on the encapsulation ratio of Ns in the nanoparticles and weight of Ns released from the microparticles.

The encapsulation ratio and encapsulation efficiency were determined using the following equations:

Encapsulation ratio [%]:

(1) $$\frac{\mathrm{Weight}\;\mathrm{of}\;\mathrm{Ns}\;\mathrm{or}\;\mathrm{Mb}\;\mathrm{in}\;\mathrm{the}\;\mathrm{nanoparticles}\;\mathrm{or}\;\mathrm{microparticles}\;\left[\mathrm{mg}\right]}{\mathrm{Weight}\;\mathrm{of}\;\mathrm{the}\;\mathrm{nanoparticles}\;\mathrm{or}\;\mathrm{microparticles}\;\left[\mathrm{mg}\right]}\times 100$$

Encapsulation efficiency [%]:

(2) $$\frac{\mathrm{Weight}\;\mathrm{of}\;\mathrm{Ns}\;\mathrm{or}\;\mathrm{Mb}\;\mathrm{in}\;\mathrm{the}\;\mathrm{nanoparticles}\;\mathrm{or}\;\mathrm{microparticles}\;\left[\mathrm{mg}\right]}{\mathrm{Weight}\;\mathrm{of}\;\mathrm{Ns}\;\mathrm{or}\;\mathrm{Mb}\;\left(\mathrm{mg}\right)}\times 100$$

The retention ratio and efficiency were determined using the following equations:

Retention ratio [%]:

(3) $$\frac{\mathrm{Weight}\;\mathrm{of}\;\mathrm{Ns}\;\mathrm{in}\;\mathrm{the}\;\mathrm{microparticles}\;\left[\mathrm{mg}\right]}{\mathrm{Microparticle}\;\mathrm{weight}\;\left[\mathrm{mg}\right]\times \left(\mathrm{encapsulation}\;\mathrm{ratio}\;\mathrm{of}\;\mathrm{Ns}\;\mathrm{in}\;\mathrm{the}\;\mathrm{nanoparticles}\;\left[\%\right]\right)/100\;}\times 100\;$$

Retention efficiency [%]:

(4) $$\frac{\mathrm{Weight}\;\mathrm{of}\;\mathrm{Ns}\;\mathrm{in}\;\mathrm{the}\;\mathrm{microparticles}\;\left[\mathrm{mg}\right]}{\mathrm{Weight}\;\mathrm{of}\;\mathrm{the}\;\mathrm{Ns}\;\left[\mathrm{mg}\right]\times \left(\mathrm{encapsulation}\;\mathrm{ratio}\;\mathrm{of}\;\mathrm{Ns}\;\mathrm{in}\;\mathrm{the}\;\mathrm{nanoparticles}\;\left[\%\right]\right)/100}\times 100$$

### 2.5. Nanoparticle and Microparticle Ns- and Mb-Release Profiles

The profiles depicting the release of Ns and Mb from the nanoparticles and microparticles were constructed using a dialysis method (*n* = 3) according to a previous report [33]. CS(Ns) or PLL(Ns) nanoparticles (1 mg), and CRG(CS(Ns), Mb) or CRG(PLL(Ns), Mb) microparticles (3 mg) were dispersed in PBS solution (1 and 3 mL, respectively). The nanoparticle (1 mL) or microparticle (3 mL) dispersion was dialyzed against PBS solution (39 and 37 mL, respectively) through a Spectra/Por6 dialysis membrane (molecular weight cut-off: 10,000; Spectrum Houston, TX, USA). Ns or Mb was released from the nanoparticles and the microparticles in a sustained manner under gentle stirring. Each sample was collected periodically (1 mL/15 min) from the exterior of the dialysis membrane and the same amount of PBS (1 mL) was added to the solution. The particle-release behavior was evaluated using three temperature patterns: (1) constant at 10 °C, (2) constant at 70 °C, and (3) ramped from 10 °C to 70 °C at 1 h after the start of experiment. The following values were determined by spectrofluorometry: (1) release ratio of Ns from the CS (Ns) or PLL (Ns) nanoparticles and (2) release ratio of Mb from the CRG(CS(Ns), Mb) or CRG(PLL(Ns), Mb) microparticles. All the releases experiments were performed three times.

## 3. Results and Discussion

### 3.1. Preparation and Characterization of the CS(Ns) and PLL(Ns) Nanoparticles

Controlling the charge states of both molecules in a solution is important when forming CS(Ns) and PLL(Ns) nanoparticles through electrostatic interactions between cationic polymers and TPP. Most of the amino groups of CS are positively charged at approximately pH 4.43 because its pK_a_ is approximately 6.5 [17,34], whereas 80% of its amino groups are deprotonated at pH 7.14 [17]. Meanwhile, three of the five phosphate groups of TPP are negatively charged at pH 4–5 [17]. Therefore, the CS nanoparticles were formed by electrostatic interactions in acetate buffer solution at pH 5. In contrast, because PLL has a pK_a_ of ~10 [35,36,37], most of its amino groups are positively charged, even at approximately pH 7. In addition, because four of the five phosphate groups of TPP are negatively charged at pH 7 [17], TPP exhibited stronger electrostatic interactions with PLL than with CS, resulting in the formation of nanoparticles with higher structural stability. However, strong electrostatic PLL–TPP interactions may also promote cross-linking reactions between nanoparticles to form nanoparticle aggregates. Therefore, the aqueous TPP solution was adjusted to pH 4 during particle formation to reduce electrostatic interactions between PLL and TPP and inhibit aggregate formation; the solution was shifted to pH 7 after particle formation to facilitate crosslinking within each nanoparticle.

**Figure 2:**
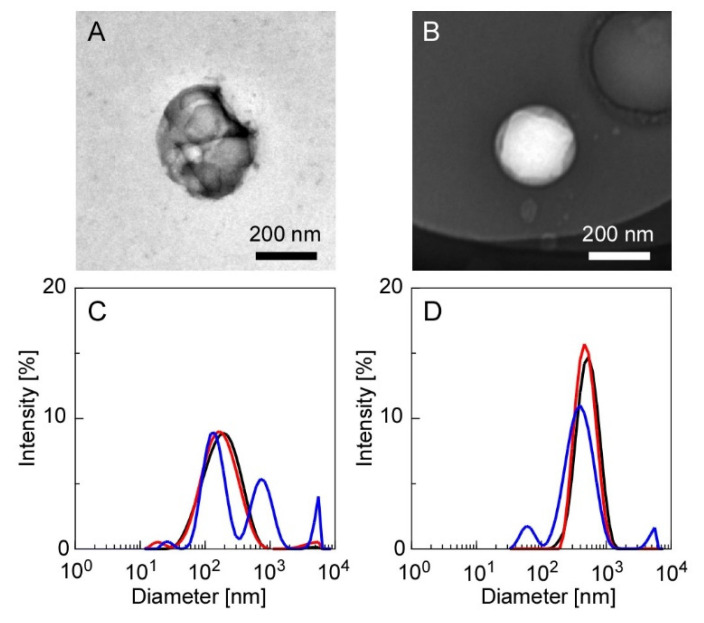
TEM images of the CS(Ns) and PLL(Ns) nanoparticles, and their respective diameter distributions evaluated by DLS ((**A**,**C**): CS(Ns) nanoparticles and (**B**,**D**): PLL(Ns) nanoparticles). Measurement conditions: 25 °C (black trace), 70 °C (red trace), and 70 °C after homogenization at 12,000 rpm for 5 min (blue trace).

Figure 2 shows the TEM images of the CS(Ns) and PLL(Ns) nanoparticles and their respective diameter distributions evaluated by DLS. Figure 2A,B display the TEM images before heating and agitation. The diameters of the CS(Ns) and PLL(Ns) nanoparticles are almost equal (100–200 nm). The particles appeared white when negatively stained (as observed for the PLL nanoparticle in Figure 2B); however, they appear black (as observed for CS nanoparticle in Figure 2A) owing to the particle overlap caused by aggregation. The aggregates of the CS(Ns) nanoparticles were attributed to the nanoparticle dialysis with Milli-Q water during nanoparticle preparation, i.e., the solution becomes almost neutral during dialysis, which lowers the positive charge of CS through progressive deprotonation and, consequently, finally aggregates the nanoparticles through hydrophobic interactions. In contrast, almost no aggregates were observed for the PLL(Ns) nanoparticles, which can be ascribed to the positive charge of PLL, even at an almost neutral pH, which prevents the formation of aggregates through electrostatic repulsion between nanoparticles. As obtained by the DLS, the diameters of the CS(Ns) and PLL(Ns) nanoparticles are approximately 200 and 500 nm, respectively, which are larger than those measured by TEM. This difference is attributed to the nanoparticle swelling in water, which is consistent with previous reports whereby nanoparticles formed from water-soluble polymers swelled and increased in size when dispersed in water [38]. Because the PLL(Ns) nanoparticles are more hydrophilic than the CS(Ns) nanoparticles, they swell more easily; hence, the DLS data show that the PLL(Ns) nanoparticles are larger (Figure 2D) than the CS(Ns) nanoparticles (Figure 2C).

To evaluate the effects of temperature and homogenization on the particle state during nanoparticle/microparticle complexation, we measured the particle diameters after heating (70 °C) and homogenization (12,000 rpm, 5 min), the results of which are shown in Figure 2C,D. The diameters of the CS(Ns) nanoparticles were relatively constant under heating; however, the diameters increased under heating and homogenization. Meanwhile, the average particle diameter of the PLL(Ns) nanoparticles did not vary significantly upon heating or homogenization. The different results suggest the higher structural stability of the PLL-TPP nanoparticles than the CS-TPP nanoparticles, which can be attributed to several factors. This includes the high electrostatic interaction between PLL and TPP. When the cross-linking reaction inside the PLL particles was carried out at pH 7, the four phosphate groups of TPP are in their ionized state. Meanwhile, when the cross-linking reaction inside the CS nanoparticles was carried out at pH 5, the three phosphate groups are in the ionized state. Another factor is the large molecular weight of PLL, which is four times that of CS, resulting in the intense entanglement of the PLL chains. The thermal stability of nanoparticles has been discussed to date [39]. Except for inorganic nanoparticles with high thermal conductivity, heat can affect the structural stability of nanoparticles.

In the absence of Ns encapsulation, the CS(-) and PLL(-) nanoparticles had average diameters of 150 and 300 nm, respectively (Figure S2). However, as shown in Figure 2, the diameters of the CS(-) nanoparticles increased from 150 nm to 200 nm, whereas those of the PLL(-) nanoparticles increased from 300 nm to 500 nm during the encapsulation of Ns. Nanoparticle size has been reported to depend on the amount of TPP added. In addition, electrostatic interactions between the cationic polymers and TPP decrease as the amount of added TPP decreases, resulting in an increase in the nanoparticle diameter [40]. Hence, in the present experimental system, the diameters of the obtained nanoparticles increased owing to the stronger electrostatic interactions between the positively charged polymers and Ns and concurrent weaker electrostatic interactions with TPP from the encapsulation of negatively charged Ns. In other words, we suggest that the nanoparticle size can easily be adjusted by changing the amount of inclusion or TPP added.

### 3.2. Ns-Release Profiles of the CS(Ns) and PLL(Ns) Nanoparticles

**Figure 3:**
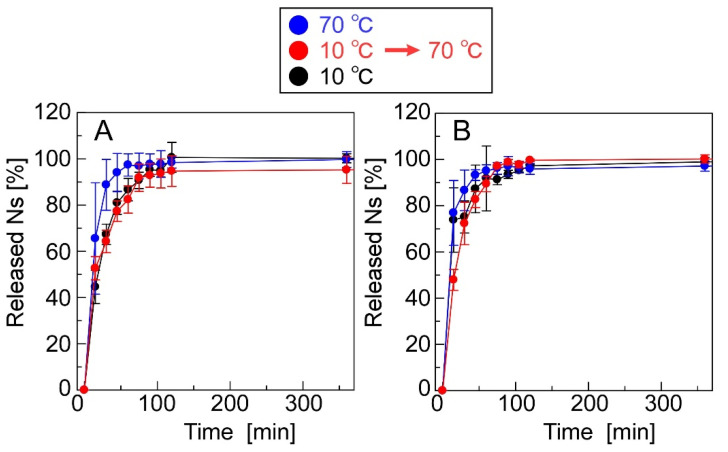
Ns-release profiles of (**A**) CS(Ns) and (**B**) PLL(Ns) nanoparticles.

Figure 3 shows Ns-release profiles of the CS(Ns) and PLL(Ns) nanoparticles, which reveals that the Ns-release behavior does not significantly vary based on the type of cationic polymer. The number of fluorescent substances (Ns or Mb) contained in each particle was determined from the fluorescence intensity of the solution at saturation because it was difficult to completely collapse the particles. The nanoparticles were formed by the electrostatic interaction of cationic polymers and anionic TPPs. Since the increase in temperature increases the mobility of each molecule, the structural stability of the particles was decreased, thus facilitating the release of the inclusions. After the number of Ns released reached saturation, increasing the temperature did not further release Ns, suggesting that there was no residual Ns retained in the polycation by intermolecular interactions (i.e., the saturated amount of Ns released is the amount of Ns that was encapsulated in the particles). Both the CS(Ns) and PLL(Ns) nanoparticles released almost 100% of their inclusions within 1 h, demonstrating that these nanoparticles rapidly released Ns. Furthermore, the CS(Ns) nanoparticle dialysis membrane dispersion became cloudy with further increases in time, whereas the analogous dispersion remained transparent for the PLL(Ns) nanoparticles.

We calculated the Ns-nanoparticle encapsulation ratios and efficiencies of CS(Ns) and PLL(Ns) (Table S1). The encapsulation ratios of CS(Ns) and PLL(Ns) were 19.6 ± 4.8% and ad 42.8 ± 5.9%, respectively. The results show that the PLL(Ns) nanoparticles encapsulated more Ns than the CS(Ns) nanoparticles, which is attributed to the different cationic properties of CS and PLL. In particular, the pH of the CS(Ns) nanoparticle dispersion is close to neutral owing to the dialysis during the preparation of the nanoparticles, and the amino groups of CS are partially deprotonated. Therefore, there was a weak interaction between CS and the negatively charged Ns, which resulted in a lower internalization ratio. The encapsulation efficiency of 33.3 ± 8.1% and 37.2 ± 5.1% for the CS(Ns) and PLL(Ns) nanoparticles, respectively, did not vary based on the type of cationic polymer used to form the nanoparticles.

### 3.3. Surface Morphologies of the CRG(CS(Ns), Mb) and CRG(PLL(Ns), Mb) Microparticles

**Figure 4:**
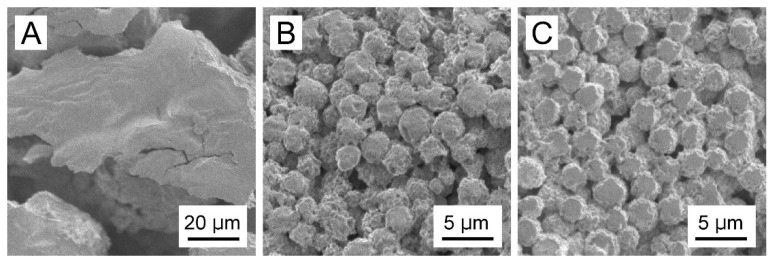
SEM images of (**A**) the white solid formed from PLL(Ns) nanoparticles devoid of CRG, (**B**) CRG(CS(Ns), Mb) microparticles, and (**C**) CRG(PLL(Ns), Mb) microparticles.

Figure 4 shows the SEM images of the white solid formed from the PLL(Ns) nanoparticles in the absence of CRG, CRG(CS(Ns), Mb) microparticles, and CRG(PLL(Ns), Mb) microparticles. No spherical structures were obtained by cooling the w/o emulsion formed from water with dispersed PLL(Ns) nanoparticles only (Figure 4A). However, microparticles were formed when the w/o emulsion prepared using a nanoparticle dispersion in a CRG solution was cooled (Figure 4B,C). We confirmed that these particles were formed by the sol–gel transition of CRG by cooling the w/o emulsion in the presence of CRG only. We measured the zeta potential of the particles under the conditions where the particles did not aggregate because the surfaces of the particles significantly affect their zeta potential [41]. The zeta potentials of the CS(Ns) and PLL(Ns) nanoparticles were determined to be 38.5 ± 1.1 and 44.9 ± 0.7 mV, respectively, whereas those of the CRG(Mb), CRG(CS(Ns), Mb), and CRG(PLL(Ns), Mb) microparticles were −38.1 ± 1.5, −34.8 ± 2.3, and −34.5 ± 1.6 mV, respectively. These values are more positive than the zeta potential of the CRG(-) microparticles (−44.1 ± 0.78 mV) reported in a previous paper [27], suggesting that the positively charged nanoparticles and Mb affects the surface properties of the microparticles. Particles with rough surfaces can be delivered more easily to deep lung sites, such as the alveoli, compared to smooth particles [42]. The rough surface of the particles causes the boundary layer on the upstream side of the particle to change from laminar to turbulent. The turbulent boundary layer can remain attached to the particle surface much longer than a laminar boundary with less eddies and, hence, creates a narrower low-pressure wake with a reduced pressure drag. The reduction in pressure drag causes the particle to travel further [43]. Thus, the rough microparticles obtained in this study are expected to be useful drug carriers for pulmonary drug-delivery applications.

### 3.4. Nanoparticle Inclusions in the Microparticles

**Figure 5:**
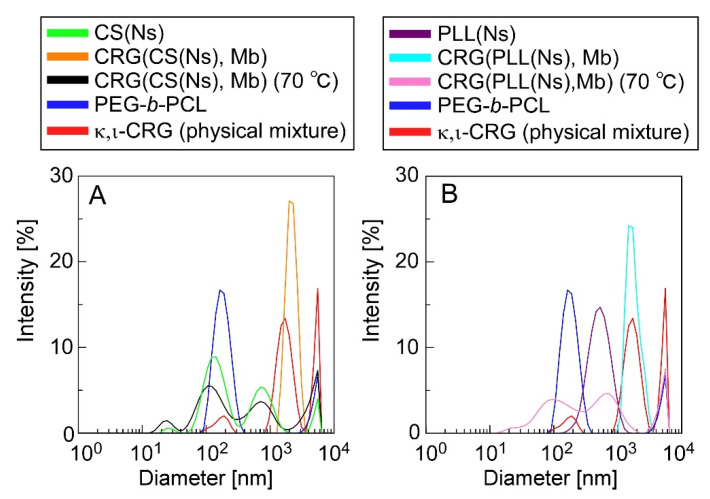
DLS-determined diameter distributions of (**A**) CRG(CS(Ns), Mb) and (**B**) CRG(PLL(Ns), Mb) microparticles. DLS data for CS (Ns) and PLL(Ns) nanoparticles, PEG-*b*-PCL, and a κ,ι-CRG (physical mixture of κ-CRG and ι-CRG) are also shown for comparison.

Figure 5 shows the DLS data for the microparticle dispersions before and after heating (70 °C), a PEG-b-PCL dispersion, an aqueous κ,ι-CRG (i.e., a physical mixture of κ-CRG and ι-CRG) solution, and a nanoparticle dispersion. The DLS data revealed that the microparticles have significantly different diameters before and after heating. The CRG(CS(Ns), Mb) microparticles (Figure 5A) exhibited a unimodal peak at 2 µm (orange trace), whereas three peaks at 100 nm, 600 nm, and 9 µm were observed after heating (black trace). Three situations are depicted in Figure 5A: (1) PEG-b-PCL was not completely dissolved when dispersed in water and formed structures with diameters of 100–200 nm (blue trace); (2) the CS(Ns) nanoparticles formed aggregates with diameters of approximately 600 nm when heated and homogenized (green trace), as shown in Figure 2; and (3) the CRG precipitated and formed structures of several micrometers in diameter after the particles were collapsed by heat because of the insolubility of CRG in water at 25 °C (red trace). Thus, the multiple peaks, which were not observed in the pre-heated CRG(CS(Ns), Mb) microparticles, were obtained after heating, owing to the PEG-b-PCL, CS(Ns) nanoparticles (and their aggregates) and CRG. Hence, despite the lack of homogenization after heating, the CS(Ns) nanoparticles aggregated owing to the presence of anionic polysaccharides. These results strongly suggest that CS(Ns) nanoparticles are complexed inside the CRG(CS(Ns), Mb) microparticles because a peak corresponding to the CS(Ns) nanoparticles was observed for the heated CRG(CS(Ns), Mb) microparticles.

In comparison, the PLL(CS(Ns), Mb) microparticles (Figure 5B) exhibited a unimodal peak at approximately 2 µm (light blue trace), and three peaks at 100 nm, 500 nm, and 9 µm (pink trace), similar to the CRG(CS(Ns), Mb) microparticles. The peaks near 100 nm and above 9 µm can be ascribed from structures formed by the PEG-b-PCL and CRG precipitates, whereas the peak at approximately 500 nm agreed well with the particle size distribution of the PLL(Ns) nanoparticles (purple trace). These results strongly suggest that the PLL(Ns) nanoparticles were complexed inside the CRG(PLL(Ns), Mb) microparticles.

**Figure 6:**
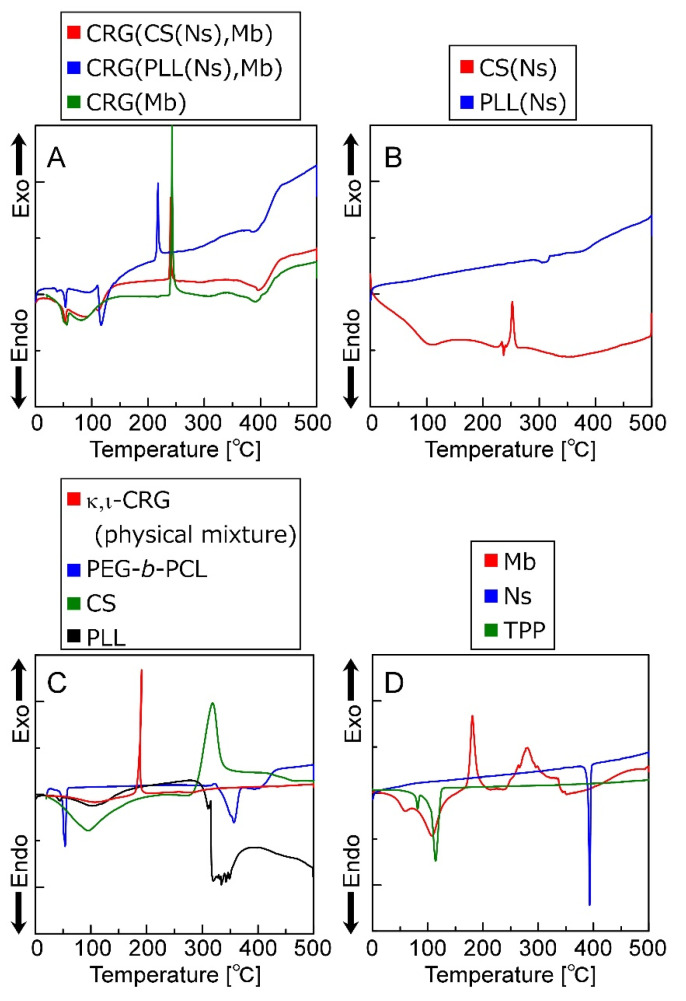
DSC thermograms (0–500 °C) of (**A**) microparticles, (**B**) nanoparticles, (**C**) polymers that form the particles and (**D**) low-molecular-weight molecules that form the microparticles and were encapsulated in the particles.

Figure 6 shows the thermal behavior of the particles, particle-forming agents, and inclusions evaluated by DSC. Figure 6 reveals that the CRG(CS(Ns), Mb), CRG(PLL(Ns), Mb), and CRG(Mb) microparticles exhibit endothermic peaks at ~55 °C, and exothermic peaks at 220 or 240 °C. Furthermore, the CRG(CS(Ns), Mb) and CRG(PLL(Ns), Mb) microparticles show minor endothermic peaks at approximately 400 °C. In contrast, the CS(Ns) nanoparticles exhibit an exothermic peak at 250 °C, whereas the PLL(Ns) nanoparticles show a minor endothermic peak at approximately 300 °C. Figure 6 also shows that PEG-b-PCL is associated with the endothermic peaks at 55 and 350 °C. In addition, κ,ι-CRG and CS exhibit exothermic peaks at 200 and 300 °C, respectively, whereas PLL exhibits an endothermic peak at ~300 °C. Mb has exothermic peaks at 200 and 300 °C. Meanwhile, Ns has an endothermic peak at 400 °C, and TPP is associated with the endothermic peaks at 90 and 120 °C.

The CS(Ns) and PLL(Ns) nanoparticles do not exhibit endothermic peaks derived from Ns (~400 °C), suggesting that Ns is uniformly dispersed inside the nanoparticles; however, the microparticles composited with these nanoparticles exhibit a minor endothermic peak at approximately 400 °C, which suggests that a small number of Ns was released from the nanoparticles, which formed local microcrystals during microparticle preparation.

The exothermic peak corresponding to the CRG particles is shifted to a higher temperature than that of the physical mixture of CRGs, indicating that CRG is more thermally stabilized by particle formation, which is ascribed to the nanoparticle internalization and the Mb inside the CRG microparticles. Pure CS has been reported to have an exothermic peak at approximately 300 °C, whereas pure PLL has an endothermic peak at approximately 300 °C [44]. The exothermic peak reportedly shifts to a higher temperature when CS and PLL are mixed with CRG owing to the electrostatic interactions between the cationic polymers and CRG [45,46]. Furthermore, Mb exhibits an exothermic peak at approximately 280 °C and is more thermally stable than CRG. Therefore, both nanoparticle complexation and electrostatic interactions between Mb and CRG can reduce the free volume of CRG and limit polymer-chain mobility, thereby improving the thermal stability of CRG. Furthermore, an endothermic peak associated with the evaporation of the water retained by each material was observed at approximately 100 °C. Therefore, these results show that microparticles several micrometers in diameter can be prepared by the emulsion method while controlling the sol–gel transition of CRG.

### 3.5. Ns- and Mb-Release Profiles of the CRG(CS(Ns), Mb) and CRG(PLL(Ns), Mb) Microparticles

**Figure 7:**
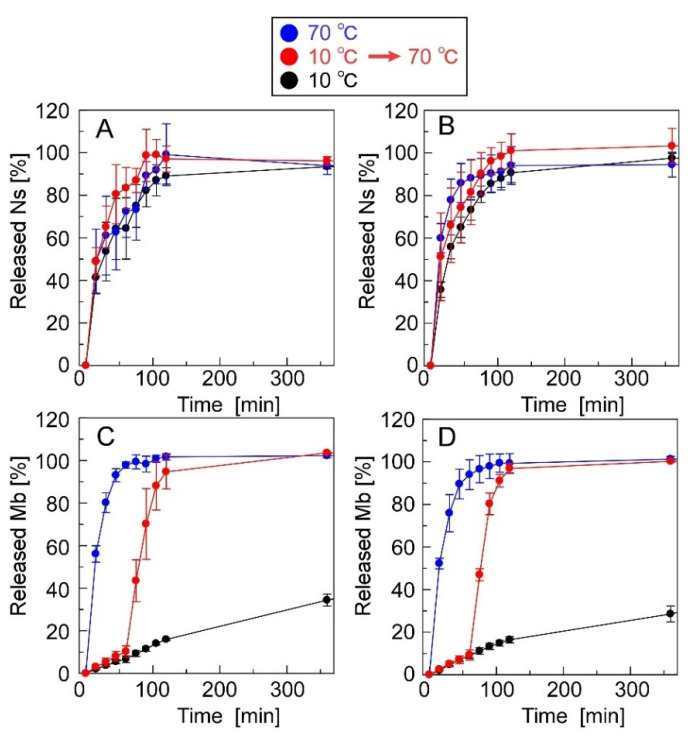
Ns- and Mb-release profiles of the microparticles. The microparticles used were (**A**,**C**) CRG(CS(Ns), Mb) and (**B**,**D**) CRG(PLL(Ns), Mb) microparticles.

Figure 7 shows the release profiles of Ns and Mb from the CRG(CS(Ns), Mb) and CRG(PLL(Ns), Mb) microparticles. Approximately 80% of Ns was released by the CRG(CS(Ns) Mb) microparticles at 60 min, whereas the CRG(PLL(Ns), Mb) microparticles released almost 100% at 120 min; both release profiles were found to be temperature-independent (Figure 7A,B). The nanoparticle complexation within the microparticles slightly reduced the Ns-release ratio because the nanoparticles released almost 100% of the Ns in 60 min (Figure 3). These results suggest that the inclusions are rapidly released from the nanoparticles, even near body temperature. In contrast, the release of Mb dispersed within the microparticles was temperature-responsive (Figure 7C,D).

We determined the retention ratios and efficiencies of the CS(Ns) and PLL(Ns) nanoparticles within the CRG(CS(Ns), Mb) and CRG(PLL(Ns), Mb) microparticles (Table S2). In addition, we analyzed the encapsulation ratios and efficiencies of Mb within the microparticles (Table S3). The type of the encapsulated nanoparticles has minimal effect on the retention ratios and efficiencies of the nanoparticles or compounds within the microparticles. The nanoparticles complexed more efficiently with the negatively charged microparticles, despite the positive charge of nanoparticles and Mb, which is attributed to the polymer-chain entanglement and electrostatic interactions during microparticle complexation. In other words, the interactions between the cationic nanoparticles and anionic CRG and the entanglement of the CRS chains from the sol–gel transition inhibited the nanoparticle release from the microparticles, resulting in a highly efficient nanoparticle/microparticle complexation.

**Figure 8:**
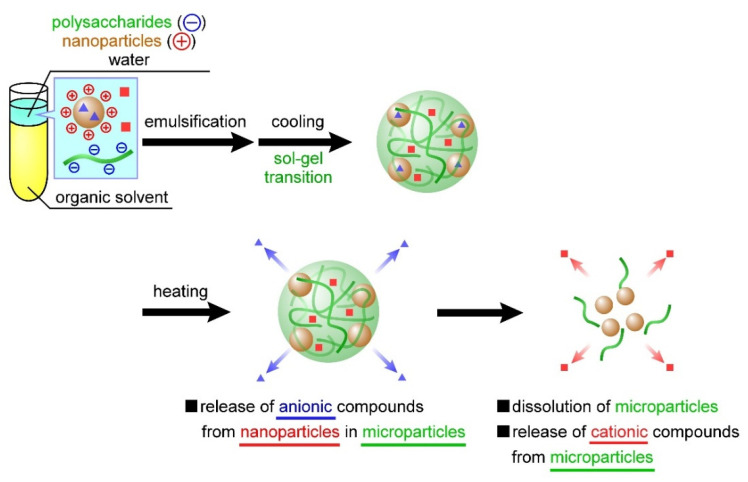
Release behavior of temperature-responsive nanoparticle-containing polysaccharide microparticles based on the experimental results of this study.

At the beginning of our research, we designed microparticles containing nanoparticles to achieve a two-step release behavior. It was assumed that Mb retained in the microparticles would be released quickly, followed by the release of Ns retained in the nanoparticles, as shown in Figure 1. In this study, a uniform dispersion of the nanoparticles inside the microparticles was assumed. However, contrary to the initial assumption, we observed the reverse order of Mb and Ns release; that is, Ns retained in the nanoparticles were released quickly, followed by the release of Mb retained in the microparticles. This difference can be attributed to the non-uniformity of the nanoparticles inside the microparticles, their presence near the surface of the microparticles, or their exposure to the solvent. Several factors can cause our above assumption concerning the coexisting structures of the nanoparticles and microparticles, namely (1) crystallinity of PEG-b-PCL, (2) surface morphologies of the particle, and (3) release properties of the particles. Although Ns was hardly released from the particles during their preparation by dialysis, Ns was rapidly released in the PBS solution (Figure 3), suggesting that the state of the nanoparticle-forming compounds (polycation and Ns) was greatly affected by the salts (Na^+^ or Cl^−^) in the PBS solution. This is mainly ascribed to the electrostatic interaction of the amino group (NH_3_^+^) in the polycationic molecule and sulfate group (SO_3_^−^ of Ns with the Na^+^ and Cl^−^ ions in the PBS solution, thereby reducing the electrostatic interaction between polycation and Ns, resulting in the rapid release of Ns from the particles. In contrast, the release of Mb retained in the microparticles was suppressed even in the PBS solution (Figure 7). As PEG-b-PCL crystallizes at approximately 20 °C [47,48], the release of Mb was suppressed at 10 °C because of the crystallization of PEG-b-PCL oriented on the surface of the microparticles, which inhibited solvent flow into the particles, thereby releasing Mb after the collapse of the particles as the temperature increased. As Ns was rapidly released from the nanoparticles without the protective effect of the PEG-b-PLC crystallization, the nanoparticles are likely located near the particle surface, or a part of the particle is exposed to the solvent. In fact, the diameter of the nanoparticles and microparticles make it difficult to uniformly disperse the nanoparticles inside the microparticles. Considering the uneven surface of the microparticles observed in Figure 4, a part of the nanoparticles surface is exposed to the solvent owing to the slower release rate of Ns from the nanoparticles in the microparticles (Figure 7) than that of the nanoparticles alone (Figure 3). Therefore, the results obtained in the present study revealed that the prepared polysaccharide microparticles containing nanoparticles can release two different charged compounds in a stepwise manner (Figure 8) with the observed release behavior different from that assumed at the beginning of this study (Figure 1).

## 4. Conclusions

In this study, we developed polysaccharide microparticles containing nanoparticles for the temperature-responsive and two-step release of inclusions. The CS nanoparticles, PLL nanoparticles, and CRG microparticles separately complexed with both nanoparticles were prepared. The inclusion ability of the nanoparticles in the microparticles, and the effect of the type of cationic polymer on the nanoparticle and microparticle release behavior were evaluated. Nanoparticle/microparticle complexation was confirmed by DSL and DSC. The nanoparticles released their inclusions rapidly regardless of the type of cationic polymer and temperature. Meanwhile, the inclusions within the nanoparticles on the microparticle surfaces were released first, after which the microparticle inclusions were released through microparticle collapse, which revealed the ability of the prepared microparticles to release two differently charged compounds in two steps.

The major challenge for future research involves including the nanoparticles uniformly within the microparticles. The process presented in the paper is expected to result in the formation of nanoparticles even with the use of various other polymers, such as polyethyleneimine (p*K*_a_: 7.0) and polyarginine (p*K*_a_: 12.0). The hydrophilicity of the nanoparticles in the solution can be adjusted significantly by judiciously choosing a polymer with the required p*K*_a_. Increasing the hydrophilicity of the nanoparticles or increasing the amount of nanoparticles and surfactants is expected to disperse the nanoparticles into the emulsion. In addition, further reduction of the nanoparticle size is another major approach for the uniform dispersion inside the microparticles. The nanoparticles are more likely to be dispersed inside the microparticles by further reducing the size of the nanoparticles by varying the ultrasound irradiation time and intensity, type of polycation, and concentration of polycation and TPP (electrostatic interaction). These approaches should promote the two-step release behavior of the microparticles in which the microparticles collapse in response to temperature, followed by the nanoparticle release and the subsequent release of their inclusions.

The particles developed in this study can release a variety of compounds independently of their electric charge, and thus have potential applications in drug delivery systems through various routes of administration, such as transpulmonary, intramuscular, and transdermal routes. In other words, these particles are expected to be used as temperature-responsive drug carriers and for the controlled release of multiple drugs. Although various further optimization studies need to be performed in order to fabricate particles that show temperature responsiveness at practical temperatures (around 37 °C), we believe that the fundamental results obtained in this paper (i.e., the ambitious challenge of two-step release using a combination of nanoparticles and microparticles) will make a significant contribution to research in the field of drug delivery systems.

## Supplementary Materials

The following supporting information can be downloaded at: https://www.mdpi.com/article/10.3390/ma15134717/s1, Figure S1: Chemical structures of the polymers (CRGs, CS, and PLL) and compound (TPP) used to form particles, and their inclusions (Mb and Ns); Figure S2: DLS-determined diameter distributions of CS(-) and PLL(-) nanoparticles; Table S1: Encapsulation ratios and encapsulation efficiencies for Ns in CS(Ns) and PLL(Ns) nanoparticles; Table S2: Retention ratios and retention efficiencies for CS(Ns) and PLL(Ns) nanoparticles inside CRG(CS(Ns), Mb) and CRG(PLL(Ns), Mb) microparticles; Table S3: Encapsulation ratios and encapsulation efficiencies of the Mb inside CRG(CS(Ns), Mb) and CRG(PLL(Ns), Mb) microparticles.
