# Supplementary material for: Temperature-Responsive Polysaccharide Microparticles Containing Nanoparticles: Release of Multiple Cationic/Anionic Compounds
Source: Materials (Basel). 2022 Jul 5;15(13):4717. doi: 10.3390/ma15134717 (PMC9268494; doi:10.3390/ma15134717)
Supplement: Supplementary file 1 [file materials-15-04717-s001.zip › materials-1781005-supplementary.pdf]

Supplementary material for

Temperature-Responsive Polysaccharide Microparticles Containing Nanoparticles: Release of Multiple Cationic/Anionic Compounds

Takumi Sato, Yoshihiko Murakami*

Department of Organic and Polymer Materials Chemistry, Faculty of Engineering, Tokyo University of Agriculture and Technology

*Corresponding author

Department of Organic and Polymer Materials Chemistry, Faculty of Engineering, Tokyo University of Agriculture and Technology, 2-24-16 Naka-cho, Koganei, Tokyo 184-8588, Japan

E-mail address: muray@cc.tuat.ac.jp (Y. Murakami)

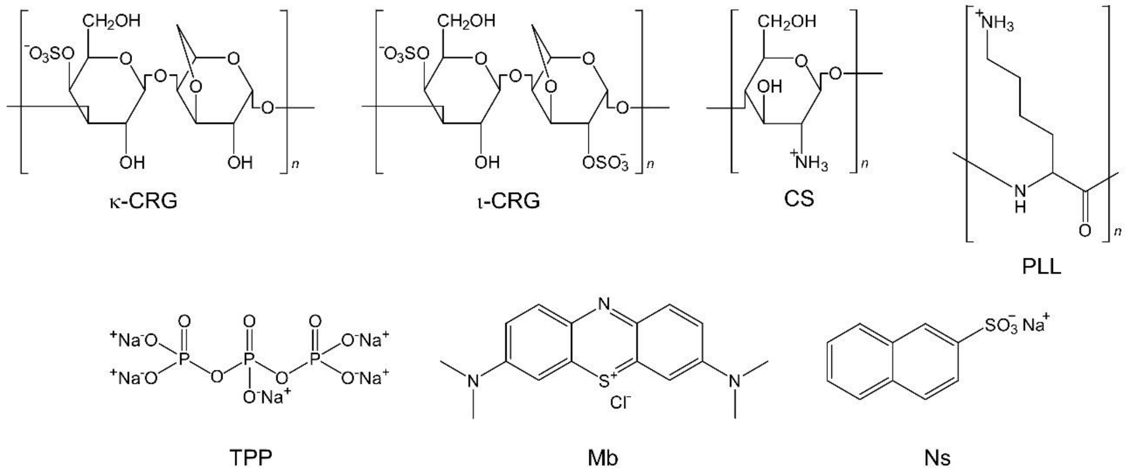

Figure S1. Chemical structures of the polymers (CRGs, CS, and PLL) and compound (TPP) used to form particles, and their inclusions (Mb and Ns).

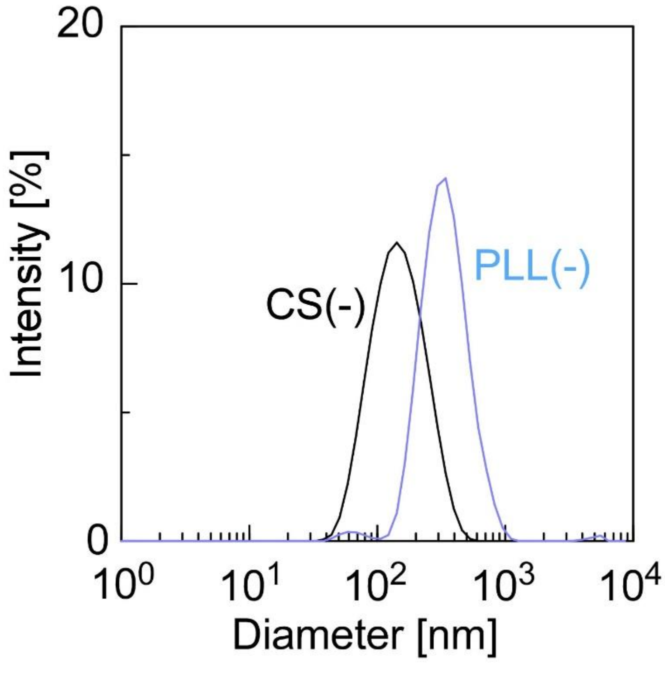

Figure S2. DLS-determined diameter distributions of CS(-) and PLL(-) nanoparticles.

Table S1. Encapsulation ratios and encapsulation efficiencies for Ns in CS(Ns) and PLL(Ns) nanoparticles.

Nanoparticle	Encapsulation ratio [%]	Encapsulation efficiency [%]
CS(Ns)	19.6 ± 4.8	33.3 ± 8.1
PLL(Ns)	42.8 ± 5.9	37.2 ± 5.1

Table S2. Retention ratios and retention efficiencies for CS(Ns) and PLL(Ns) nanoparticles inside CRG(CS(Ns), Mb) and CRG(PLL(Ns), Mb) microparticles

Microparticle	Nanoparticle	Retention ratio [%]	Retention efficiency [%]
CRG(CS(Ns), Mb)	CS(Ns)	0.31 ± 0.03	51.6 ± 10.1
CRG(PLL(Ns), Mb)	PLL(Ns)	0.087 ± 0.04	60.8 ± 11.0

Table S3. Encapsulation ratios and encapsulation efficiencies of the Mb inside CRG(CS(Ns), Mb) and CRG(PLL(Ns), Mb) microparticles

Microparticle	Encapsulation ratio [%]	Encapsulation efficiency [%]
CRG(CS(Ns), Mb)	0.49 ± 0.04	15.6 ± 2.5
CRG(PLL(Ns), Mb)	0.44 ± 0.01	13.7 ± 0.69
